# Role of comorbidities and medication on immunotherapy efficacy in cSCC: A DeCOG multicentre analysis

**DOI:** 10.1111/jdv.70307

**Published:** 2026-01-29

**Authors:** Glenn Geidel, Sabrina Bänsch, Laura Adam, Nathan Fekade, Alessandra Rünger, Tim Zell, Daniel J. Smit, Julian Kött, Isabel Heidrich, Michael Weichenthal, Selma Ugurel, Ulrike Leiter, Peter Mohr, Alexandra Geusau, Stephan Grabbe, Markus V. Heppt, Ralf Gutzmer, Jochen Utikal, Jan‐Christoph Simon, Sebastian Haferkamp, Laura Susok, Bastian Schilling, Edgar Dippel, Imke von Wasielewski, Jessica C. Hassel, Carola Berking, Stefan W. Schneider, Christoffer Gebhardt

**Affiliations:** ^1^ Department of Dermatology and Venerology University Medical Center Hamburg‐Eppendorf Hamburg Germany; ^2^ Fleur Hiege Center for Skin Cancer Research University Medical Center Hamburg‐Eppendorf Hamburg Germany; ^3^ Institute of Tumor Biology University Medical Center Hamburg‐Eppendorf Hamburg Germany; ^4^ Department of Dermatology University Hospital of Schleswig‐Holstein Kiel Germany; ^5^ Department of Dermatology Medical School and University Medical Center OWL, Klinikum Bielefeld Rosenhöhe, Bielefeld University Bielefeld Germany; ^6^ Division of Dermatooncology, Department of Dermatology University Medical Center Tuebingen Germany; ^7^ Department of Dermatology and Venerology Elbe Klinikum Buxtehude Buxtehude Germany; ^8^ Department of Dermatology Medical University of Vienna Vienna Austria; ^9^ Department of Dermatology University Medical Center of the Johannes Gutenberg‐University Mainz Mainz Germany; ^10^ Department of Dermatology, Uniklinikum Erlangen Friedrich‐Alexander‐University Erlangen‐Nürnberg Erlangen Germany; ^11^ Department of Dermatology, Venereology, Allergology and Phlebology, Johannes Wesling Klinikum, Mühlenkreiskliniken AöR University Medical Center of the Ruhr University Bochum Minden Germany; ^12^ Department of Dermatology, Venereology and Allergology, University Medical Center Mannheim Ruprecht‐Karl University of Heidelberg Mannheim Germany; ^13^ Skin Cancer Unit German Cancer Research Center (DKFZ) Heidelberg Germany; ^14^ DKFZ Hector Cancer Institute at the University Medical Center Mannheim Mannheim Germany; ^15^ Department of Dermatology Venereology and Allergology, University Medical Center Leipzig Leipzig Germany; ^16^ Department of Dermatology and Venereology University Medical Center Regensburg Regensburg Germany; ^17^ Department of Dermatology, Klinikum Dortmund gGmbH University Witten/Herdecke Dortmund Germany; ^18^ Department of Dermatology Goethe University Frankfurt, University Hospital Frankfurt am Main Germany; ^19^ Department of Dermatology Skin Tumor Center, Klinikum Ludwigshafen gGmbH Ludwigshafen Germany; ^20^ Department of Dermatology, Venerology and Allergology, Skin Cancer Center Hannover University Hospital Hannover Medical School Hannover Germany; ^21^ Department of Dermatology and National Center for Tumor Diseases (NCT), NCT Heidelberg a Partnership between DKFZ and University Hospital Heidelberg Medical Faculty Heidelberg, Heidelberg University Heidelberg Germany

**Keywords:** anticoagulants, comorbidity, haematologic neoplasms, immune checkpoint inhibitors, immunosuppressive agents, multicentre study, skin neoplasms, squamous cell carcinoma

## Abstract

**Background:**

Immune checkpoint inhibitors (ICI) have transformed the treatment landscape of advanced cutaneous squamous cell carcinoma (cSCC). The influence of comorbidities and concomitant medications on treatment efficacy remains incompletely defined.

**Objectives:**

To evaluate the impact of comorbid conditions and commonly prescribed medications on progression‐free survival (PFS) and overall survival (OS) in patients with advanced cSCC receiving ICI.

**Methods:**

In this multicentre cohort study, 273 patients with unresectable or metastatic cSCC treated with ICI were identified from the prospective ADOReg skin cancer registry. Data on comorbidities, such as haematologic malignancies, immunosuppressive conditions, cardiovascular disease and concomitant medications, such as immunosuppressive agents and anticoagulants, were analysed. Treatment outcomes were measured as PFS and OS.

**Results:**

Among first‐line patients (*n* = 253), immunosuppressive conditions were associated with shorter PFS (5.5 vs. 24.4 months, *p* = 0.012) and OS (16.6 vs. 34.1 months, *p* < 0.001). Haematologic malignancies were likewise linked to poorer PFS (18.6 vs. 27.0 months, *p* = 0.032) and OS (17.0 vs. 33.6 months, *p* = 0.0067). Concomitant anticoagulant therapy correlated with longer PFS (49.3 vs. 17.4 months, *p* = 0.032), but not OS (*p* = 0.22). In multivariate analysis, immunosuppressive disease remained independently associated with shorter OS (HR = 9.88, 95% CI: 1.11–87.5, *p* = 0.040) and anticoagulant use with longer PFS (HR = 0.34, 95% CI: 0.15–0.80, *p* = 0.014). These associations were confirmed in Cox models weighted by inverse probability of treatment (IPTW), supporting robustness to confounding. No effect was attributable to any specific anticoagulant subclass.

**Conclusions:**

Our analyses suggest that immunosuppressive disease is an independent predictor of shortened OS, underscoring the critical role of host immune competence. We further report a novel, independently significant association between anticoagulant use and prolonged PFS. Other associations should be regarded as exploratory. These findings highlight host‐related factors as potential modulators of ICI efficacy and merit prospective validation.

## Plain Language Summary

**What is the Disease?:**

Cutaneous squamous cell carcinoma (cSCC) is a common type of skin cancer. In advanced stages, when surgery is no longer possible, treatment with immune checkpoint inhibitors (ICI) has become a standard of care option.

**Location of the Study:**

This research was conducted across multiple dermatology centres in Germany, Austria and Switzerland as part of a collaborative study group (DeCOG).

**Aim of the Study:**

We wanted to understand how other health conditions and commonly used medications may influence the success of ICI treatment in patients with advanced cSCC.

**Methodology:**

Medical records of 273 patients with advanced cSCC treated with ICI were analysed. We looked at survival outcomes and compared them across groups with different health conditions and medications.

**Key Findings:**

Patients with diseases that weaken the immune system responded less well to ICI, and this effect remained significant even after accounting for other factors. Blood cancers also showed an association with poorer outcomes, but this did not remain significant after adjustment. In contrast, patients taking medications that prevent blood clots (anticoagulants) had longer periods without cancer progression, and this association remained significant even after adjustment. The effect was observed for anticoagulant use overall, but no specific drug class (such as aspirin, newer oral anticoagulants or heparins) could be singled out. Other common medications had no clear impact on treatment outcomes.

**Conclusions and Practical Implications:**

Certain health conditions and medications may influence how well immunotherapy works in advanced cSCC. These insights could help doctors better assess risks and guide personalized treatment decisions. Further studies are needed to confirm these observations.

## Graphical abstract

Immunosuppressive disease was associated with reduced overall survival, while anticoagulant use was linked to prolonged progression‐free survival in advanced cutaneous squamous cell carcinoma treated with immune checkpoint inhibitors. Host‐related factors may modulate immunotherapy efficacy and warrant prospective validation. Created in BioRender. Geidel, G. (2025) https://BioRender.com/9vzpqnh.

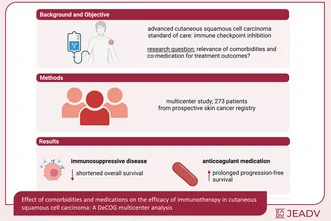

**Why was the study undertaken**

- The efficacy of immune checkpoint inhibitors (ICIs) in advanced cutaneous squamous cell carcinoma (cSCC) is well established, but data on the impact of comorbidities and concomitant medications on treatment outcomes are scarce. This study was undertaken to systematically assess how patient‐related clinical factors influence progression‐free and overall survival in a large, real‐world cSCC cohort receiving ICI therapy.

**What does this study add**

- Immunosuppressive disease emerged as an independent predictor of poorer survival, while anticoagulant use showed a novel and independently significant association with improved progression‐free survival. Haematologic malignancies were associated with worse outcomes in univariate analyses but did not retain independent significance. Importantly, the effect of anticoagulants was observed at the overall level, with no single subclass accounting for the association.

**What are the implications of this study for disease understanding and/or clinical care**

- This study underscores the relevance of host‐related factors on the outcome of immunotherapy in patients with advanced cSCC. It highlights the need for individualized risk stratification and suggests that widely used medications, such as anticoagulants, may influence the effectiveness of ICI. These findings are hypothesis‐generating and call for prospective validation, mechanistic investigation and integration of clinical context into future treatment algorithms.

## INTRODUCTION

Cutaneous squamous cell carcinoma (cSCC) is the second most common skin cancer and accounts for a growing proportion of skin cancer–related mortality, particularly in elderly and immunocompromised populations.^1^, ^2^ In recent years, immune checkpoint inhibitors (ICI), such as PD‐1 inhibitors, have become the standard of care for advanced, unresectable or metastatic cSCC, demonstrating high response rates and durable tumour control in trials and real‐world cohorts.^3^, ^4^, ^5^, ^6^

ICI outcomes vary between patients, prompting interest in host‐specific factors influencing treatment efficacy.^7^, ^8^, ^9^, ^10^ Comorbidities such as haematologic malignancies or immunosuppressive conditions are common in patients with advanced cSCC and may alter antitumor immunity.^11^ In contrast to other tumour entities, data on the role of concomitant medications in modulating ICI efficacy in cSCC remain scarce. Some drugs, such as hydrochlorothiazide, have been linked to cSCC carcinogenesis, while mTOR inhibitors are preferred over other immunosuppressants in organ transplant recipients due to a lower cSCC risk. The impact of concomitant medications on immunotherapy outcomes remains underexplored in this population.^12^, ^13^, ^14^ Understanding these associations is relevant due to the high prevalence of polypharmacy and comorbidities in elderly cSCC patients. Despite these considerations, data on the prognostic relevance of comorbidities and co‐medications in cSCC immunotherapy are sparse. A systematic analysis in a large, real‐world population has so far been lacking.

In this multicentre cohort study, we aimed to comprehensively assess the impact of comorbidities and concomitant medications on progression‐free survival (PFS) and overall survival (OS) in patients with advanced cSCC treated with ICI therapy. Particular focus was given to immunosuppressive conditions, haematologic malignancies and commonly prescribed medications to identify clinically relevant associations and potential hypotheses for future prospective investigations.

## METHODS

### Study design

We retrospectively analysed 273 patients with histologically confirmed, unresectable or metastatic cSCC who received ICI therapy in different treatment lines and were enrolled in the prospective multicentre ADOReg skin cancer registry of the German Dermatologic Cooperative Oncology Group (DeCOG) between 2016 and 2024. All patients had documented follow‐up. In addition, available records on comorbidities and concomitant medications were reviewed. ICIs included PD‐1 inhibitors (cemiplimab, pembrolizumab and nivolumab) and combination regimens (ipilimumab/nivolumab). The registry was approved by the Ethics Committee of the University of Duisburg‐Essen (14–5921‐BO), and all patients gave written informed consent.

### Comorbidities and concomitant medications

Comorbidities were coded according to ICD‐10, the dominant classification system used across Germany and Europe and applied within the ADOReg registry, ensuring consistent and structured documentation. For analysis, ICD‐10 codes were grouped into broader, clinically meaningful categories at different hierarchical levels (chapter, block, three‐digit code). In addition, clinically meaningful categories were established. ‘Immunosuppression’ included the use of immunomodulatory medication (corticosteroids, azathioprine, mycophenolate mofetil, calcineurin inhibitors, JAK inhibitors, methotrexate and cladribine) and/or diseases associated with an immunosuppressive status (ICD B15–B24, C81–C96, D45 and D80–D90). The narrower category ‘immunosuppressive disease’ referred to these conditions alone. ‘Cardiovascular disease’ comprised comorbidities affecting the cardiovascular system (defined as: Q20–Q28, I05‐15, I20‐25, I35, I42, I44‐50, I63, I64, I70‐71, I74 and G45.9). ‘Haematological malignancy’ included ICD C81–C96. Concomitant medications were recorded at the substance level, grouped into pharmacological classes and broader categories. ‘Anticoagulant therapy’ included platelet aggregation inhibitors, direct oral anticoagulants (DOACs), low‐molecular‐weight heparins (LMWH) and vitamin K antagonists (VKAs). Hydrochlorothiazide, antibiotics and metformin were analysed separately, given their previously described potential relevance in cSCC or immunotherapy.

### Definition of clinical outcomes

Treatment outcomes included objective response rate (ORR), disease control rate (DCR), progression‐free survival (PFS) and overall survival (OS). Response was assessed as complete response (CR), partial response (PR), stable disease (SD) or progressive disease (PD).^15^ ORR comprised CR and PR; DCR comprised CR, PR or SD. PFS was defined as the time from ICI initiation to progression, death of any case or last follow‐up. OS was defined as the time from ICI initiation to death of any case or last follow‐up.

### Statistical analysis

Clinical and demographic characteristics were summarized descriptively. Group comparisons used Pearson's chi‐squared test with Yates' correction, Wilcoxon rank‐sum test or Fisher's exact test, as appropriate. Survival probabilities were estimated with the Kaplan–Meier method and compared with the two‐sided log‐rank test.

When survival curves crossed and log‐rank tests were significant, the proportional hazards assumption was tested using the Grambsch–Therneau method based on Schoenfeld residuals. Where applicable, time‐dependent Cox models were applied using tt() with log(*t* + 0.01).

Multivariate Cox proportional hazards models were performed to identify potential independent markers of interest. Covariates included demographic (age, sex), clinical (AJCC stage, Eastern Cooperative Oncology Group (ECOG) performance status, high‐risk localization, metastasis pattern, radiotherapy status), comorbidity‐related (immunosuppression, immunosuppressive disease, haematologic malignancy) and medication‐related variables (anticoagulant therapy and hydrochlorothiazide). To assess the robustness of selected associations, sensitivity analyses using inverse probability of treatment weighting (IPTW) Cox models were performed. Propensity scores were estimated via logistic regression using relevant baseline covariates and used to calculate inverse probability weights. Statistical analyses were conducted using Python in Google Colab, R (RStudio version 2024.09.0) and GraphPad Prism (version 10.4.0). All *p*‐values were two‐sided, and values <0.05 were considered statistically significant.

## RESULTS

### Cohort characteristics

**FIGURE 1:**
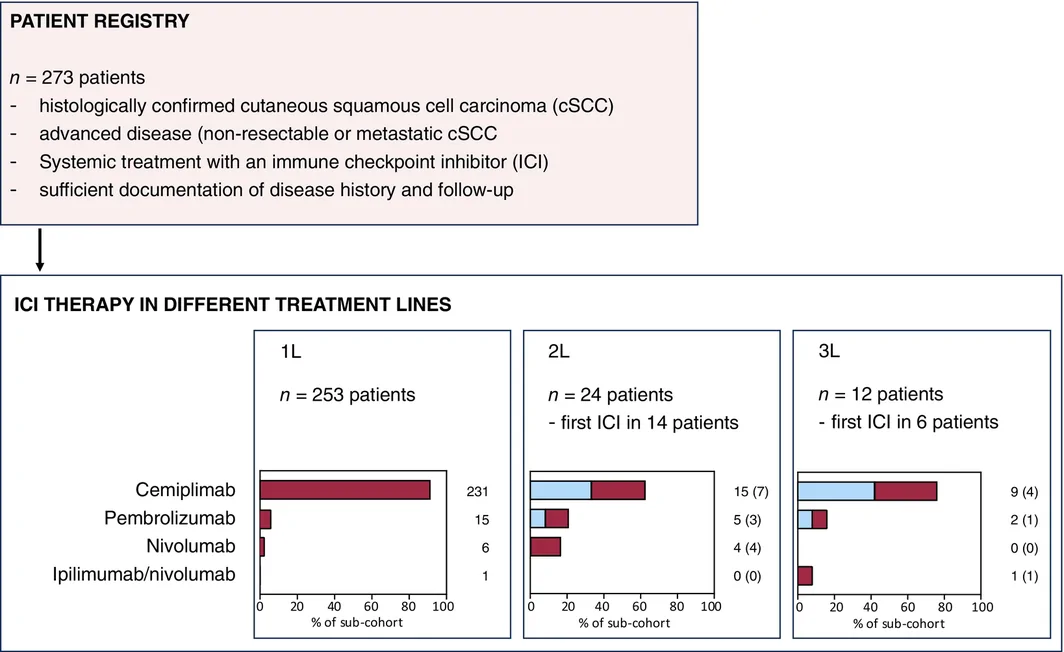
Cohort allocation. Graphical illustration of the allocation process of advanced cSCC patients receiving immunotherapy (ICI) from the registry. Treatment lines (1L, 2L, 3L) with respective sub‐cohorts are stratified by type of ICI administered. Black bars reflect patients receiving ICI for the first time, whereas grey bars depict patients receiving ICI after previous treatment with ICI. The total number of patients receiving the respective ICI treatments is indicated. Bracketed numbers indicate the number of patients with the first ICI, where applicable.

A total of 273 patients with histologically confirmed advanced cSCC were included in the analysis (Figure 1). Most received ICI in the first‐line setting (*n* = 253, 92.7%), while only small subsets were treated in the second‐ (*n* = 24, 8.8%) or third‐line setting (*n* = 12, 4.4%). The characteristics and outcomes of patients treated with ICI beyond the first‐line are summarized in the Supplementary Material (Figures S1–S6, Table S1). Among first‐line patients, the median age at diagnosis was 80 years (IQR: 74–85), and the majority were male (84.2%) (Table S2). ECOG performance status was 0–1 in 47.8% of cases. High‐risk anatomical sites such as the ear, lip or temple were involved in 21.3% of patients. At treatment initiation, 17% presented with distant metastases, 34.4% with locoregional spread and 48.6% had no evidence of metastasis (Table S2).

### Comorbidities and concomitant medications

**FIGURE 2:**
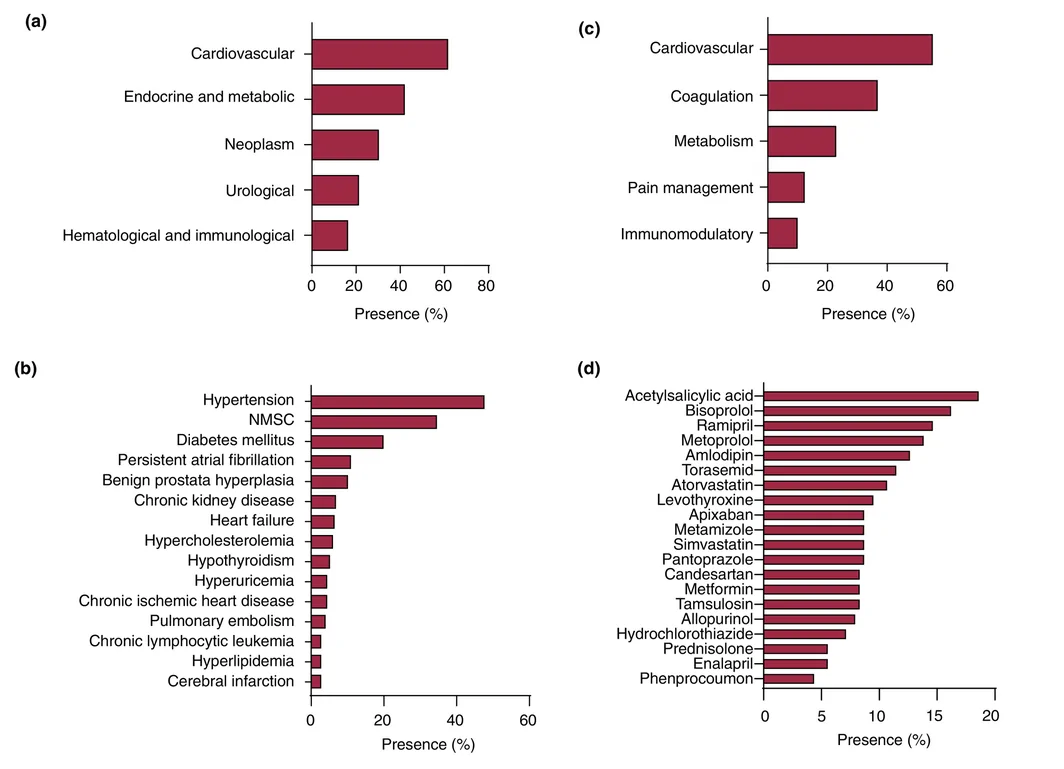
Distribution of comorbidities and concomitant medications. (a) Presence of top 5 comorbidity classes in per cent from total patients. (b) Presence of top 15 diagnoses in the cohort in per cent from total patients. (c) Presence of top 5 medication groups in per cent of total patients. (d) Presence of top 20 substances in the cohort in per cent of total patients. ICD‐10, International Classification of Diseases, 10th revision; NMSC, non‐melanoma skin cancer.

Cardiovascular comorbidities were most common (61.6%), followed by endocrine/metabolic disorders (42.0%) and other neoplasms (30.2%) (Figure 2a). The high frequency of neoplasms was largely driven by additional non‐melanoma skin cancers, including further cSCC (Figure 2b). Haematologic and immunologic conditions, such as chronic lymphocytic leukaemia (CLL), were present in 16.3% of patients (Figure 2a, Figure S7).

Cardiovascular and metabolic medications predominated among concomitant treatments (55.3% and 22.9%, respectively), reflecting the comorbidity profile (Figure 2c). Anticoagulant therapy was documented in 36.8% of patients. Among these, acetylsalicylic acid (as the most frequent antiplatelet agent) and apixaban (as the most frequent direct oral anticoagulant) were most commonly prescribed (Figure 2d). Immunomodulatory agents were used in 9.9%, and hydrochlorothiazide, previously linked to increased NMSC risk, was prescribed in 7.1% (Figure 2d, Figure S8).

### Association of comorbidities with clinical outcomes

**FIGURE 3:**
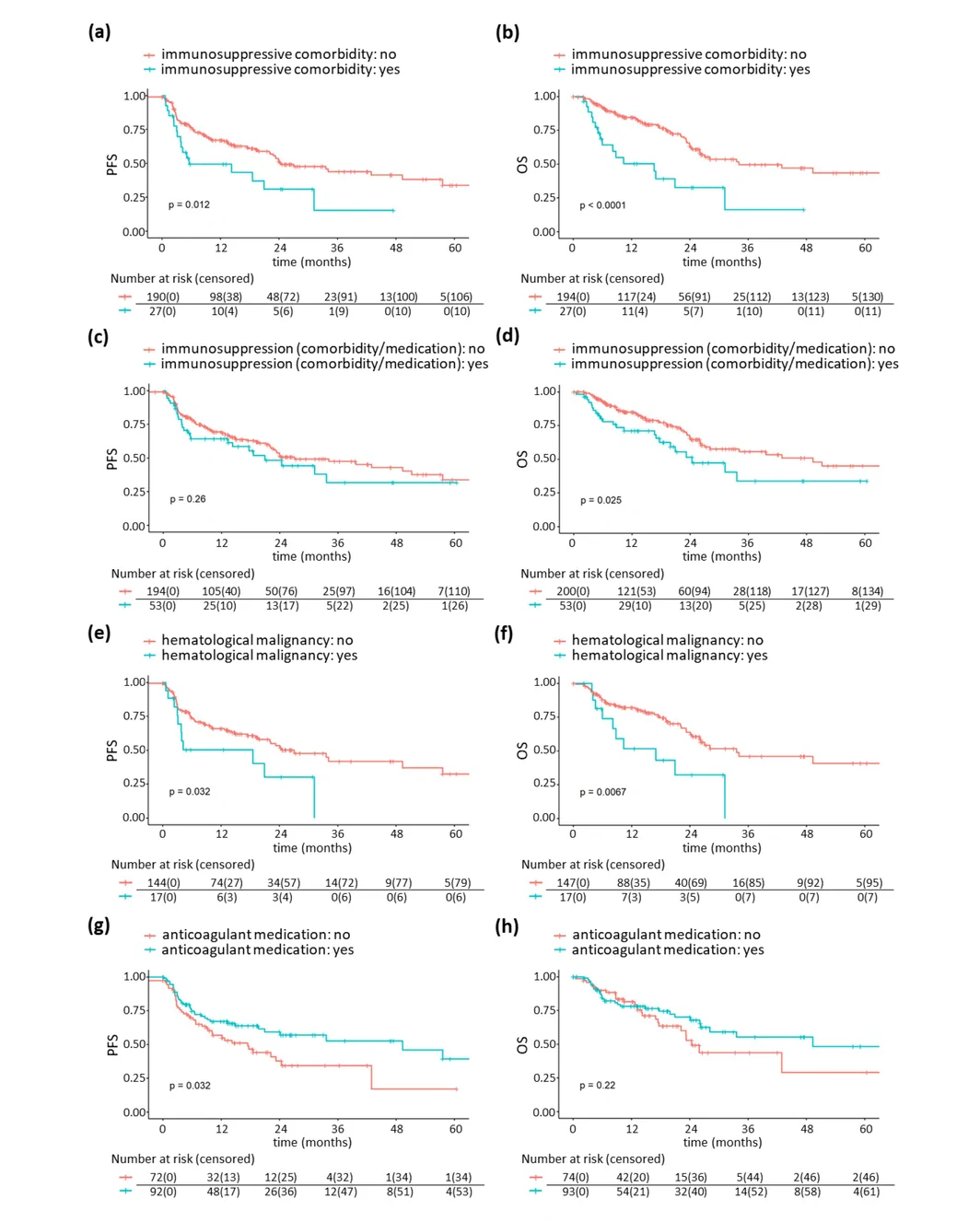
Outcome of first‐line treatment stratified by immunologic comorbidities. Analysis of PFS and OS in first‐line immunotherapy of cSCC for the categories of (a, b) immunosuppressive comorbidity overall, (c, d) immunosuppressive state due to comorbidity or intake of immunosuppressant medication, (e, f) haematologic malignancy and (g, h) anticoagulant concomitant medication. *P* values were calculated using the log‐rank test. The number of patients at risk at each time point is shown; values in parentheses represent the cumulative number of censored patients up to that time point.

Given the high prevalence of comorbid conditions in this elderly patient population, we next evaluated their potential impact on clinical outcomes. Stratification by ICD‐10 chapter did not reveal significant associations with PFS or OS (Figures S9 and S10). By contrast, clinically defined categories were informative: patients with immunosuppressive diseases had significantly shorter PFS (5.5 vs. 24.4 months, *p* = 0.012) and OS (16.6 vs. 34.1 months, *p* < 0.001) (Figure 3a,b, Table S2). The broader category of immunosuppression, which also included immunomodulatory medication, was associated with reduced outcomes, reaching statistical significance for OS (*p* = 0.025) but not for PFS (*p* = 0.26) (Figure 3c,d, Table S2).

Haematologic malignancies were likewise linked to poorer survival (PFS: 18.6 vs. 27.0 months, *p* = 0.032; OS: 17.0 vs. 33.6 months, *p* = 0.0067) (Figure 3e,f, Table S2). In contrast, outcomes did not differ significantly in patients with organ transplants (Figure S11a,b) or in those with CLL, the most frequent haematologic malignancy in the cohort (Figure S11c,d).

In multivariate analysis, immunosuppressive disease remained independently associated with reduced OS (HR: 9.88, 95% CI: 1.11–87.5, *p* = 0.040), whereas haematologic malignancy did not emerge as an independent predictor (Table 1). Other clinical parameters, including AJCC stage and ECOG performance status, were not significantly associated with survival in the adjusted model. Radiotherapy was included as a covariate given its clinical relevance, but did not suggest a significant independent association with PFS or OS (Table 1). This association was confirmed in an IPTW‐weighted analysis, in which immunosuppressive disease again showed a significant association with shortened OS (HR: 3.86, 95% CI: 1.43–10.43, *p* = 0.008).

**TABLE 1:** Multivariate Cox proportional hazards analysis in first‐line ICI‐treated cSCC patients.

	PFS		OS	
	HR (CI)	*p*‐value	HR (CI)	*p*‐value
Anticoagulatory medication	**0.34 (0.15–0.80)**	**0.014**	0.45 (0.18–1.14)	0.093
Haematological malignancy	0.12 (0.01–1.30)	0.082	0.11 (0.01–1.18)	0.068
Immunosuppressive disease	5.89 (0.69–50.03)	0.10	**9.88 (1.11–87.5)**	**0.040**
Sex (male)	2.53 (0.82–7.78)	0.10	1.65 (0.49–5.62)	0.42
ECOG (high)	1.39 (0.87–2.22)	0.15	1.56 (0.94–2.58)	0.087
Immunosuppression	0.62 (0.12–3.10)	0.30	0.75 (0.15–3.81)	0.72
Radiation (yes)	2.26 (0.27–18.85)	0.45	0.50 (0.03–8.85)	0.63
Hydrochlorothiazide	1.41 (0.35–5.73)	0.63	0.45 (0.09–2.39)	0.35
AJCC stage (high)	1.04 (0.71–1.51)	0.84	1.06 (0.69–1.61)	0.80
Age (high)	1.00 (0.93–1.06)	0.88	0.97 (0.90–1.03)	0.32

*Note*: Multivariable Cox regression analysis evaluating the association of clinical variables, comorbidities and selected concomitant medications with progression‐free survival (PFS) and overall survival (OS) in patients receiving immune checkpoint inhibitor (ICI) therapy in first‐line treatment of advanced cutaneous squamous cell carcinoma (cSCC). Hazard ratios (HR) with 95% confidence intervals (CI) and *p*‐values are shown for PFS and OS. Bold font indicates statistically significant values.

### Association of concomitant medications with clinical outcomes

To assess the role of concomitant medications, we first examined the four most prevalent drug groups as well as the five most frequently prescribed individual agents, none of which showed significant associations with survival (Figures S12–S14). By contrast, anticoagulant use was linked to longer PFS (49.3 vs. 17.4 months, *p* = 0.032), while OS was unaffected (*p* = 0.22) (Figure 3g,h, Table S2). Stratification by anticoagulant subclass did not reveal significant subgroup differences (Figure S15). Given reports implicating hydrochlorothiazide in cSCC development, we evaluated its association with ICI outcomes. PFS was shorter in hydrochlorothiazide users (*p* = 0.036), whereas OS was unaffected (*p* = 0.61) (Figure S16a,b). Antibiotic therapy, previously linked to impaired ICI efficacy in other cancers, showed no significant impact (Figure S16c,d). Given preclinical data suggesting antitumor effects of metformin in cSCC models, we also evaluated concomitant metformin therapy.^16^ Metformin use was not associated with differences in PFS or OS in our cohort (Figure S16e,f).

In multivariate analysis, anticoagulant use remained independently associated with prolonged PFS (HR: 0.34, 95% CI: 0.15–0.80, *p* = 0.014), while hydrochlorothiazide, antibiotics and metformin did not retain statistical significance (Table 1). This association was confirmed in an IPTW‐weighted analysis, where anticoagulant use was again significantly associated with prolonged PFS (HR: 0.37, 95% CI: 0.18–0.77, *p* = 0.008).

## DISCUSSION

In this multicentre, real‐world analysis, we systematically evaluated the prognostic relevance of comorbidities and concomitant medications in patients with advanced cSCC treated with ICI. Leveraging a prospectively documented registry cohort characterized by high comorbidity burden and comprehensive clinical annotation, we identified distinct host‐related factors that may inform stratification and therapeutic decision‐making.

Consistent with prior evidence, both haematologic malignancies and immunosuppressive conditions were associated with shorter progression‐free survival and overall survival.^11^ The prognostic impact was particularly pronounced in patients with immunosuppressive disease, such as lymphoproliferative or chronic infectious disorders, whereas pharmacologic immunosuppression alone was associated with more moderate outcome differences. These findings reflect varying degrees of immune impairment and underscore the importance of distinguishing disease—from medication‐related immunosuppression when evaluating responsiveness to ICI.^17^ In multivariate analysis, the presence of immunosuppressive disease remained an independent predictor of reduced overall survival (HR: 9.88, 95% CI: 1.11–87.5, *p* = 0.040), emphasizing the profound prognostic impact of underlying immune dysfunction. Haematologic malignancies, in contrast, did not retain independent significance and should currently be regarded as an exploratory signal. Given a limited number of patients in this sub‐analysis, which may have introduced bias, further data will be crucial to clarify the prognostic role of haematological malignancies. This is particularly relevant for cSCC, a tumour entity strongly driven by impaired immune surveillance.^18^, ^19^

A novel observation in our cohort was the association of concomitant anticoagulant medication with longer PFS. This effect was observed at the overall level of anticoagulant use and remained significant in multivariate analysis, suggesting an independent association. Stratification within drug subclasses (antiplatelet agents, DOACs, LMWH and VKAs) did not identify a single category driving this effect. Thus, while the overall signal of prolonged PFS is robust, the present data do not indicate that it can be attributed to a specific subclass of anticoagulant medication.

Anticoagulants have been implicated in modulating immune function by interfering with thrombin signalling, platelet–tumour interactions and vascular adhesion molecules, thereby shaping immune cell trafficking and effector activity in the tumour microenvironment.^20^, ^21^, ^22^, ^23^ Serum D‐dimers, a sensitive indicator of coagulation activity, have recently been identified as an independent predictive pre‐treatment biomarker in advanced cSCC patients treated with cemiplimab.^24^ Prior studies in melanoma and other cancer entities have suggested immunomodulatory effects of anticoagulants.^25^, ^26^, ^27^, ^28^, ^29^ Our findings extend these hypotheses to cSCC and highlight coagulation–immune cross‐talk as a potential modifier of ICI efficacy. Nevertheless, residual confounding by cardiovascular comorbidity, indication bias and treatment‐related frailty cannot be excluded. Prospective studies incorporating biomarker profiling and precise exposure characterization will be essential to clarify this association.

We also assessed the impact of hydrochlorothiazide, a diuretic with reported photocarcinogenic properties and epidemiologic links to cSCC risk.^30^ Interestingly, we observed a significant association with reduced PFS, though no effect was present for OS. A proportional hazards violation prompted use of a time‐dependent Cox model, which confirmed a persistent hazard increase over time (HR = 3.15, *p* = 0.0315), without a significant time interaction. This suggests a stable adverse effect, possibly due to tumour biology or selection bias. Moreover, it raises the hypothesis that prior or chronic exposure to hydrochlorothiazide may impact tumour immunogenicity or immune microenvironment priming.^31^ The widespread use of hydrochlorothiazide in elderly populations highlights the importance of further research into its long‐term oncologic implications. Notably, hydrochlorothiazide use was not significantly associated with survival outcomes in multivariate analysis, though the small sample size may have limited statistical power.

In contrast, antibiotic use showed no significant association with survival, differing from prior reports in other cancers.^32^ Differences in timing, exposure windows and limited subgroup size may account for this discrepancy. Given the complexity of host–microbiome–tumour interactions, this remains an important area for mechanistic study.^33^ Likewise, metformin, which slowed tumour growth in a murine cSCC model, did not impact PFS or OS in our cohort.^16^ These data suggest that while experimental findings support a potential antitumor effect, larger clinical datasets will be needed to determine whether such benefits translate into the patient setting.

Beyond these specific signals, most other prevalent comorbidities and co‐medications, including cardiovascular and metabolic agents, did not show a consistent effect on ICI efficacy. This aligns with the multivariate analysis, where age, sex, AJCC stage and most evaluated comorbidities and medications did not emerge as independent predictors.

While our analysis benefits from prospective multicentre registry data, well‐defined exposure criteria and a structured analytic framework, several limitations must be acknowledged. These include the retrospective nature of the analysis, the absence of longitudinal data on medication adherence or changes during therapy, the lack of sufficiently detailed information to stratify comorbidities and concomitant medications according to disease activity or treatment status, limited statistical power in certain sub‐groups, such as patients with CLL, haematological malignancies or organ transplant recipients, as well as the small number of patients with radiotherapy (*n* = 21), which restricts the interpretability of this covariate. As with all observational studies, residual confounding cannot be excluded. Nonetheless, our data represent one of the largest real‐world cohorts of cSCC patients treated with ICI and provide clinically relevant insights that may guide future stratification strategies and trial design.

## CONCLUSIONS

This study provides a comprehensive real‐world assessment of comorbidities and concomitant medications in relation to ICI outcomes in advanced cSCC. Immunosuppressive disease emerged as an independent predictor of reduced overall survival, underscoring the critical role of host immune competence. Anticoagulant use showed a robust and independently significant association with prolonged progression‐free survival, representing a novel and clinically relevant observation that warrants prospective validation. Other associations, including those with haematologic malignancies, hydrochlorothiazide or antibiotic use, did not retain independent significance and should be regarded as exploratory. Taken together, our findings highlight selected host‐related factors as potential modulators of immunotherapy efficacy and point to the need for validation in prospective studies, stratified trial designs and mechanistic investigations.

## AUTHOR CONTRIBUTIONS

GG: conceptualization, data curation, formal analysis, investigation—data analysis, investigation—data interpretation, methodology, project administration, resources, software, supervision, validation, visualization, writing—original draft, writing—review & editing. SB, LA, NF, AR, TZ, DJS, IH, MW, UL, PM, AG, SG, MH, RF, JU, J‐CS, SH, LS, BS, ED, IW, JCH, CB, SWS: data curation, resources, writing—review & editing. SU: data curation, resources, supervision, writing—review & editing. CG: conceptualization, data curation, resources, supervision, writing—review & editing.

## FUNDING INFORMATION

The authors have nothing to report.

## CONFLICT OF INTEREST STATEMENT

GG has received honoraria from Almirall Hermal, BMS, derma2go, SUN Pharma, Janssen‐Cilag and Mylan, outside the submitted work. GG has received travel expenses from MSD and SUN Pharma, outside the submitted work. GG has received grants from Regeneron Pharmaceuticals and Delcath Systems outside the submitted work. GG is a member of the advisory board of Regeneron. GG is a board member of SCOPE, unpaid. JK has received speakers' honoraria from BMS, Sanofi and UCB; JK has received travel support from Pierre‐Fabre and SUN Pharma; all outside the submitted work. IH has received grants from the Mildred Scheel Cancer Center of the University Medical Center Hamburg‐Eppendorf, the Fleur‐Hiege Stiftung—die Deutsche Hautkrebsstiftung and the Deutsche Forschungsgemeinschaft. IH has received honoraria for lectures and educational events from Almirall, Bristol‐Myers Squibb and Sysmex Inostics; all outside the submitted work. SU declares research support from Bristol‐Myers Squibb and Merck‐Serono; speakers' and advisory board honoraria from Bristol‐Myers Squibb, Merck Sharp & Dohme, Merck‐Serono and Novartis; and meeting and travel support from Almirall, Bristol‐Myers Squibb, IGEA Clinical Biophysics, Merck Sharp & Dohme, Novartis, Pierre Fabre and Sun Pharma; outside the submitted work. UL declares research support from Merck Sharp & Dohme; speakers' and advisory board honoraria from Novartis, Sanofi, Regeneron, Sun Pharma, Almirall Hermal; and meeting and travel support from Pierre Fabre and Sun Pharma; outside the submitted work. RG has received grants from Amgen, Sanofi‐Regeneron, Merck‐Serono, Kyowa‐Kirin, Almirall, SUN Pharma, Recordati; consulting fees from Bristol‐Myers Squibb, Novartis, MSD, Almirall‐Hermal, Amgen, Pierre Fabre, Merck‐Serono, SUN Pharma, Sanofi, Immunocore, Delcath, KyowaKirin, Janssen; honoraria for lectures from Bristol‐Myers Squibb, Novartis, MSD, Almirall‐Hermal, Amgen, Merck‐Serono, SUN Pharma, Pierre Fabre, Sanofi‐Regeneron; travel support from SUN Pharma and Pierre Fabre; honoraria for advisory board participation from Bristol‐Myers Squibb, Novartis, MSD, Almirall‐Hermal, Amgen, Pierre Fabre, Merck‐Serono, SUN, Sanofi‐Regeneron, Immunocore and KyowaKirin; all outside the submitted work. JU is on the advisory board or has received honoraria and travel support from Amgen, Bristol‐Myers Squibb, GSK, Immunocore, LeoPharma, Merck Sharp and Dohme, Novartis, Pierre Fabre, Rheacell, Roche and Sanofi outside the submitted work. BS reports relevant financial activities (speakers' and advisory board honoraria from Sanofi, Pierre Fabre Pharmaceuticals, Regeneron, BMS, Immunocore, Blueprint Medicine and SUN Pharma, outside the submitted work). JCH declares speaker honoraria from BMS, Delcath, Immunocore, MSD, Novartis, Pierre Fabre, Sanofi and Sunpharma and honoraria for advisory board participation from Onkowissen and Sunpharma. CB declares speaker's and/or advisory board's honoraria from Almirall, Bristol‐Myers Squibb, Delcath, Immunocore, Merck Sharp & Dohme, Novartis, Pierre Fabre and Regeneron, outside the submitted work. CG is a member of the advisory board of BMS, Immunocore, MSD, Novartis, Pierre Fabre, Sanofi, SUN Pharma, Sysmex. CG has received honoraria by BMS, GSK, Immunocore, MSD, Novartis, Pierre Fabre, Sanofi, SUN Pharma, Sysmex, outside the submitted work. CG has received travel expenses from BMS, Pierre Fabre and SUN Pharma, outside the submitted work. CG is a board member of the DeCOG (ADO), unpaid; CG is a board member of the Hiege Stiftung, unpaid. CG is a board member of the Roggenbuck‐Stiftung, unpaid. CG is a founder of Dermagnostix and Dermagnostix R&D. All other authors declare no conflicts of interest.

## ETHICAL APPROVAL

The registry has been approved by the Medical Ethics Committee of the University Duisburg‐Essen (14‐5921‐BO).

## ETHICS STATEMENT

Not applicable.

## Supporting information


Data S1:

## Data Availability

The data that support the findings of this study are available from the corresponding authors upon reasonable request.
