# Supplementary material for: Role of comorbidities and medication on immunotherapy efficacy in cSCC: A DeCOG multicentre analysis
Source: J Eur Acad Dermatol Venereol. 2026 Jan 29;40(9):1525–34. doi: 10.1111/jdv.70307 (PMC13505687; doi:10.1111/jdv.70307)
Supplement: Supplementary file 1 [file JDV-40-1525-s001.zip › jeadv-2025-2514-File007.docx]

| Supplementary Table 1: Characteristics and outcome for second- and third-line immunotherapy stratified by comorbidities and comedication | | | | | | | | | | | | | |
| --- | --- | --- | --- | --- | --- | --- | --- | --- | --- | --- | --- | --- | --- |
|  | **All patients** | **immunosuppression** | | | **immunosuppressive**  **disease** | | | **hematological malignancy** | | | **anticoagulant medication** | | |
|  |  | IS  n (%) | no IS  n (%) | p value | ISD  n (%) | no ISD  n (%) | p value | HM  n (%) | no HM  n (%) | p value | AM  n (%) | no AM  n (%) | p value |
| Total | 36 (100) | 10 (100) | 26 (100) |  | 8 (100) | 19 (100) |  | 7 (100) | 11 (100) |  | 10 (100) | 5 (100) |  |
| ECOG |  |  |  | 0.29 |  |  | 0.15 |  |  | 0.39 |  |  | 0.41 |
| 0 | 10 | 5 (50) | 5 (19.2) |  | 5 (62.5) | 3 (15.8) |  | 4 (57.1) | 2 (18.2) |  | 3 (30) | 0 (0) |  |
| 1 | 8 | 1 (10) | 7 (26.9) |  | 1 (12.5) | 6 (31.6) |  | 1 (14.3) | 4 (36.4) |  | 3 (30) | 3 (60) |  |
| 2 | 2 | 0 (0) | 2 (7.7) |  | 0 (0) | 2 (10.5) |  | 0 (0) | 2 (18.2) |  | 2 (20) | 0 (0) |  |
| 3 | 0 | 0 (0) | 0 (0) |  | 0 (0) | 0 (0) |  | 0 (0) | 0 (0) |  | 0 (0) | 0 (0) |  |
| unknown | 16 | 4 (4) | 12 (46.2) |  | 2 (25) | 8 (42.1) |  | 2 (28.6) | 3 (27.3) |  | 2 (20) | 2 (40) |  |
| Age |  |  |  | 0.89 |  |  | 0.82 |  |  | 0.31 |  |  | 0.83 |
| median (IQR) | 74 (66-77) | 73 (69-74) | 75 (62-78) |  | 73 (69-74) | 74 (63-79) |  | 73 (69-75) | 78 (69-84) |  | 77 (67-83) | 75 (74-75) |  |
| AJCC stage |  |  |  | 0.97 |  |  | 0.14 |  |  | 0.25 |  |  | 0.20 |
| I | 10 | 2 (20) | 8 (30.8) |  | 1 (12.5) | 7 (36.8) |  | 1 (14.3) | 2 (18.2) |  | 2 (20) | 3 (60) |  |
| II | 5 | 2 (20) | 3 (11.5) |  | 2 (25) | 3 (15.8) |  | 2 (28.6) | 1 (9.1) |  | 1 (10) | 1 (20) |  |
| III | 11 | 3 (30) | 8 (30.8) |  | 3 (37.5) | 6 (31.6) |  | 2 (28.6) | 5 (45.5) |  | 5 (50) | 0 (0) |  |
| IV | 7 | 2 (20) | 5 (19.2) |  | 2 (25) | 0 (0) |  | 2 (28.6) | 0 (0) |  | 1 (10) | 0 (0) |  |
| unknown | 3 | 1 (10) | 2 (7.7) |  | 0 (0) | 3 (15.8) |  | 0 (0) | 3 (27.3) |  | 1 (10) | 1 (20) |  |
| Sex |  |  |  | 0.62 |  |  | 0.55 |  |  | 0.99 |  |  | 0.52 |
| male | 31 | 8 (80) | 23 (88.5) |  | 6 (75) | 17 (89.5) |  | 6 (85.7) | 9 (81.8) |  | 8 (80) | 5 (100) |  |
| female | 5 | 2 (20) | 3 (11.5) |  | 2 (25) | 2 (10.5) |  | 1 (14.3) | 2 (18.2) |  | 2 (20) | 0 (0) |  |
| High-risk location |  |  |  | 0.55 |  |  | 0.53 |  |  | 0.24 |  |  | 0.99 |
| yes | 4 | 0 (0) | 4 (15.4) |  | 0 (0) | 3 (15.8) |  | 0 (0) | 3 (27.3) |  | 2 (20) | 1 (20) |  |
| no | 32 | 10 (100) | 22 (84.6) |  | 8 (100) | 16 (84.2) |  | 7 (100) | 8 (72.7) |  | 8 (80) | 4 (80) |  |
| Metastases |  |  |  | 0.35 |  |  | 0.75 |  |  | 0.24 |  |  | 0.51 |
| locoregional | 13 | 2 (20) | 11 (42.3) |  | 2 (25) | 8 (42.1) |  | 1 (14.3) | 6 (54.5) |  | 6 (60) | 3 (60) |  |
| distant | 8 | 2 (20) | 6 (23.1) |  | 2 (25) | 4 (21.1) |  | 2 (28.6) | 1 (9.1) |  | 0 (0) | 1 (20 |  |
| none | 15 | 6 (60) | 9 (34.6) |  | 4 (50) | 7 (36.8) |  | 4 (57.1) | 4 (36.4) |  | 4 (40) | 1 (20) |  |
| ICI type |  |  |  | 0.73 |  |  | 0.4 |  |  | **0.011** |  |  | 0.095 |
| cemiplimab | 24 | 6 (60) | 18 (69.2) |  | 4 (50.0) | 14 (73.7) |  | 3 (42.8) | 10 (90.9) |  | 9 (90) | 3 (60) |  |
| pembrolizumab | 7 | 2 (20) | 5 (19.3) |  | 2 (25.0) | 3 (15.8) |  | 2 (28.6) | 0 (0) |  | 0 (0) | 2 (40) |  |
| nivolumab | 4 | 2 (20) | 2 (7.7) |  | 2 (25.0) | 1 (5.3) |  | 2 (28.6) | 0 (0) |  | 0 (0) | 0 (0) |  |
| ipilimumab/  nivolumab | 1 | 0 (0) | 1 (3.8) |  | 0 (0) | 1 (5.3) |  | 0 (0) | 1 (9.1) |  | 1 (10) | 0 (0) |  |
| Best Overall Response |  |  |  | 0.25 |  |  | 0.1 |  |  | **0.018** |  |  | 0.69 |
| CR | 12 | 2 (20) | 10 (38.5) |  | 1 (12.5) | 6 (31.6) |  | 0 (0) | 3 (27.1) |  | 4 (40) | 1 (20) |  |
| PR | 3 | 0 (0) | 3 (11.5) |  | 0 (0) | 3 (15.8) |  | 0 (0) | 2 (18.7) |  | 1 (10) | 0 (0) |  |
| SD | 5 | 1 (10) | 4 (15.4) |  | 0 (0) | 3 (15.8) |  | 0 (0) | 3 (27.1) |  | 1 (10) | 1 (20) |  |
| PD | 16 | 7 (70) | 9 (34.6) |  | 7 (87.5) | 7 (36.8) |  | 7 (100) | 3 (27.1) |  | 4 (40) | 3 (60) |  |
| Objective response | 15 | 2 (20) | 13 (50) | 0.2 | 1 (12.5) | 9 (47.4) | 0.2 | 0 (0) | 5 (45.4) | 0.11 | 5 (50) | 1 (20) | 0.57 |
| Disease control | 20 | 3 (30) | 17 (65.4) | 0.12 | 1 (12.5) | 12 (63.2) | **0.047** | 0 (0) | 8 (72.7) | **0.011** | 6 (60) | 2 (40) | 0.99 |
| PFS |  |  |  | **0.016** |  |  | **< 0.001** |  |  | **0.021** |  |  | 0.35 |
| median  (95% CI) | 44.5 months (6.0 – NR) | 6.4 months  (2.1 – NR) | 32.2 months  (12.3 – NR) |  | 4.6 months  (2.1 – NR) | 21.3 months  (13.4 – NR) |  | 5.0 months  (2.1 – NR) | 16.9 months  (8.7 – NR) |  | 12.3 months  (7.8 – NR) | 13.2 months  (0.9 – NR) |  |
| 1-year PFS rate  (95% CI) | 63.2%  (44.8 – 89) | 30.0%  (11.6-77.3) | 69.3%  (52.7 – 91.1) |  | 12.5%  (1.0 – 78.2) | 76.0%  (58.0 – 99.6) |  | 14.3%  (2.3 – 87.7) | 68.6%  (44.5 – 100) |  | 55.6%  (31.0 – 99.7) | 50.0%  (18.8 – 100) |  |
| OS |  |  |  | 0.2 |  |  | 0.46 |  |  | 0.16 |  |  | **0.001** |
| median  (95% CI) | 45.5 months (40.6 – NR) | 16.9 months  (13.0 – NR) | 62.2 months  (28.9 – NR) |  | 14.6 months  (13.0 – NR) | 33.6 months  (21.3 – NR) |  | 14.6 months  (5.2 – NR) | NR  (21.3 – NR) |  | NR  (NR – NR) | 14.9 months  (1.1 – NR) |  |
| 1-year OS rate  (95% CI) | 84.2%  (69.3 – 100) | 80.0%  (58.7 – 100) | 86.5%  (73.4 – 100) |  | 75.0%  (50.3 – 100) | 81.9%  (65.3 – 100) |  | 71.4%  (44.7 – 100) | 90.0%  (73.2 – 100) |  | 100%  (100 – 100) | 75.0%  (42.6 – 100) |  |
