# Supplementary material for: Role of comorbidities and medication on immunotherapy efficacy in cSCC: A DeCOG multicentre analysis
Source: J Eur Acad Dermatol Venereol. 2026 Jan 29;40(9):1525–34. doi: 10.1111/jdv.70307 (PMC13505687; doi:10.1111/jdv.70307)
Supplement: Supplementary file 1 [file JDV-40-1525-s001.zip › jeadv-2025-2514-File008.docx]

| Supplementary Table 2: Characteristics and outcome for first-line immunotherapy stratified by comorbidities and comedication | | | | | | | | | | | |  |  |
| --- | --- | --- | --- | --- | --- | --- | --- | --- | --- | --- | --- | --- | --- |
|  | **All patients** | **immunosuppression** | | | **immunosuppressive**  **disease** | | | **hematological malignancy** | | | **anticoagulant medication** | | |
|  |  | IS  n (%) | no IS  n (%) | p value | ISD  n (%) | no ISD  n (%) | p value | HM  n (%) | no HM  n (%) | p value | AM  n (%) | no AM  n (%) | p value |
| Total | 253 (100) | 53 (100) | 200 (100) |  | 27 (100) | 194 (100) |  | 17 (100) | 147 (100) |  | 93 (100) | 74 (100) |  |
| ECOG |  |  |  | 0.58 |  |  | 0.40 |  |  | 0.26 |  |  | 0.74 |
| 0 | 60 (23.7) | 16 (30.2) | 44 (22) |  | 10 (37) | 40 (20.6) |  | 7 (41.2) | 25 (17) |  | 15 (16.1) | 15 (20.3) |  |
| 1 | 61 (24.1) | 14 (26.4) | 47 (23.5) |  | 7 (25.9) | 51 (26.3) |  | 4 (23.5) | 40 (27.2) |  | 26 (28.0) | 19 (25.7) |  |
| 2 | 28 (11.1) | 6 (11.3) | 22 (11) |  | 3 (11.2) | 24 (12.4) |  | 1 (5.9) | 20 (13.6) |  | 16 (17.2) | 8 (10.8) |  |
| 3 | 6 (2.4) | 1 (1.9) | 5 (2.5) |  | 0 (0) | 6 (3.1) |  | 0 (0) | 6 (4.1) |  | 3 (3.2) | 2 (2.7) |  |
| unknown | 98 (38.7) | 16 (30.2) | 82 (41) |  | 7 (25.9) | 73 (73.6) |  | 5 (29.4) | 56 (38.1) |  | 33 (35.5) | 30 (40.5) |  |
| Age |  |  |  | **0.019** |  |  | 0.30 |  |  | 0.12 |  |  | 0.055 |
| median (IQR) | 80 (74-85) | 77 (73-82) | 80 (74-85) |  | 78 (74-82) | 80 (73-85) |  | 79 (73-82) | 80 (74-85) |  | 81 (76-85) | 79 (73-83) |  |
| AJCC stage |  |  |  | **0.014** |  |  | **0.043** |  |  | 0.093 |  |  | 0.174 |
| I | 51 (20.2) | 10 (18.8) | 41 (20.5) |  | 2 (7.4) | 42 (21.6) |  | 1 (5.9) | 28 (19) |  | 16 (17.2) | 14 (18.9) |  |
| II | 40 (15.8) | 11 (20.7) | 29 (14.5) |  | 7 (25.9) | 27 (13.9) |  | 5 (29.4) | 19 (12.9) |  | 12 (12.9) | 10 (13.5) |  |
| III | 78 (30.8) | 22 (41.5) | 56 (28) |  | 13 (48.1) | 58 (29.9) |  | 9 (52.9) | 50 (34) |  | 41 (44.1) | 20 (27) |  |
| IV | 52 (20.6) | 3 (5.6) | 49 (24.5) |  | 2 (7.4) | 42 (21.7) |  | 1 (5.9) | 29 (19.8) |  | 14 (15.1) | 19 (25.7) |  |
| unknown | 32 (12.6) | 7 (13.2 | 25 (12.5) |  | 3 (11.2) | 25 (12.9) |  | 1 (5.9) | 21 (14.3) |  | 10 (10.8) | 11 (14.9) |  |
| Sex |  |  |  | 0.20 |  |  | 0.26 |  |  | 0.078 |  |  | 0.99 |
| male | 213 (84.2) | 48 (90.6) | 165 (82.5) |  | 25 (92.6) | 160 (82.4) |  | 17 (100) | 120 (81.6) |  | 77 (82.8) | 62 (83.8) |  |
| female | 40 (15.8) | 5 (9.4) | 35 (17.5) |  | 2 (7.4) | 34 (17.6) |  | 0 (0) | 27 (18.4) |  | 16 (17.2) | 12 (16.2) |  |
| High-risk location |  |  |  | 0.70 |  |  | 0.60 |  |  | 0.99 |  |  | 0.84 |
| yes | 54 (21.3) | 10 (18.8) | 44 (22) |  | 6 (22.2) | 36 (18.6) |  | 3 (17.6) | 28 (19) |  | 17 (18.3) | 15 (20.3) |  |
| no | 199 (78.7) | 43 (81.2) | 156 (78) |  | 21 (87.8) | 158 (81.4) |  | 14 (82.4) | 119 (81) |  | 76 (81.7) | 59 (79.7) |  |
| Metastases |  |  |  | 0.41 |  |  | 0.47 |  |  | **0.034** |  |  | 0.49 |
| locoregional | 87 (34.4) | 18 (34) | 69 (34.5) |  | 12 (44.4) | 63 (32.5) |  | 9 (52.9) | 45 (30.6) |  | 31 (33.3) | 23 (31.1) |  |
| distant | 43 (17) | 6 (11.3) | 37 (18.5) |  | 3 (11.2) | 35 (18) |  | 0 (0) | 30 (20.4) |  | 18 (19.4) | 10 (13.5) |  |
| none | 123 (48.6) | 29 (54.7) | 94 (47) |  | 12 (44.4) | 96 (49.5) |  | 8 (47.1) | 72 (49) |  | 44 (47.3) | 41 (55.4) |  |
| ICI type |  |  |  | 0.73 |  |  | 0.76 |  |  | 0.99 |  |  | 0.85 |
| cemiplimab | 231 (91.3) | 50 (94.3) | 181 (90.5) |  | 25 (92.6) | 181 (93.3) |  | 16 (94.1) | 137 (93.2) |  | 86 (92.4) | 71 (94.6) |  |
| pembrolizumab | 15 (5.9) | 3 (5.7) | 12 (6) |  | 2 (7.4) | 10 (5.2) |  | 1 (5.9) | 8 (5.4) |  | 6 (6.5) | 3 (5.4) |  |
| nivolumab | 6 (2.2) | 0 (0) | 6 (3) |  | 0 (0) | 2 (1) |  | 0 (0) | 2 (1.4) |  | 1 (1.1) | 0 (0) |  |
| ipilimumab/  nivolumab | 1 (0.4) | 0 (0) | 1 (0.5) |  | 0 (0) | 1 (0.5) |  | 0 (0) | 0 (0) |  | 0 (0) | 0 (0) |  |
| Best Overall Response |  |  |  | 0.64 |  |  | 0.14 |  |  | 0.28 |  |  | 0.41 |
| CR | 69 (27.3) | 12 (22.6) | 57 (28.5) |  | 5 (18.6) | 56 (28.9) |  | 3 (17.6) | 40 (27.2) |  | 23 (24.7) | 17 (23.0) |  |
| PR | 60 (23.7) | 11 (20.8) | 49 (24.5) |  | 7 (25.9) | 42 (21.6) |  | 4 (23.5) | 30 (20.4) |  | 21 (22.6) | 13 (17.6) |  |
| SD | 57 (22.5) | 13 (24.5) | 44 (22.0) |  | 3 (11.1) | 45 (23.2) |  | 2 (11.8) | 37 (25.2) |  | 26 (28.0) | 17 (23.0) |  |
| PD | 67 (26.5) | 17 (32.1) | 50 (25.0) |  | 12 (44.4) | 51 (26.3) |  | 8 (47.1) | 40 (27.2) |  | 23 (24.7) | 27 (36.4) |  |
| Objective response | 129 (51) | 23 (43) | 106 (53) | 0.27 | 12 (44.4) | 98 (50.5) | 0.69 | 7 (41.1) | 70 (47.6) | 0.8 | 44 (47.3) | 30 (40.5) | 0.47 |
| Disease control | 186 (73.5) | 36 (68) | 150 (75) | 0.38 | 15 (56.5) | 143 (73.7) | 0.083 | 9 (52.9) | 107 (72.7) | 0.15 | 70 (75.2) | 47 (63.5) | 0.13 |
| PFS |  |  |  | 0.26 |  |  | **0.012** |  |  | **0.032** |  |  | **0.032** |
| median  (95% CI) | 24.0 months  (21.7 - 48.6) | 20.9 months  (13.3 - NR) | 27.0 months  (22.2 - 57.6) |  | 5.5 months  (3.8 – NR) | 24.4 months  (22.2 - 57.6) |  | 18.6 months  (3.8 - NR) | 27.0 months (19.7 - NR) |  | 49.3 months  (20.9 - NR) | 17.4 months  (9.2 – NR) |  |
| 1-year PFS rate  (95% CI) | 67.6%  (61.8 - 74) | 64.2%  (52.2 - 79.0) | 69.2%  (62.8 - 76.4) |  | 49.4%  (33.3 - 73.4) | 67.1%  (60.4 - 74.5) |  | 50.4%  (31.0 - 81.9) | 65.8%  (58.3 - 74.4) |  | 67.0%  (57.6 - 77.9) | 56.7%  (45.9 - 70.1) |  |
| OS |  |  |  | **0.025** |  |  | **<0.001** |  |  | **0.0067** |  |  | 0.22 |
| median  (95% CI) | 39.1 months (25.8 – NR) | 24.4 months  (17.6 - NR) | 49.2 months  (28.1 -NR) |  | 16.6 months  (5.9 - NR) | 34.1 months  (26.1 - NR) |  | 17.0 months  (8.1 – NR) | 33.6 months  (26.1 - NR) |  | 49.3 months  (28.1 - NR | 24.4 months  (22.2 – NR) |  |
| 1-year OS rate  (95% CI) | 81.9%  (77 – 87.2) | 71.1%  (59.4 - 85.2) | 84.9%  (79.7 - 90.4) |  | 50.4%  (33.7 - 75.4) | 84.6%  (79.3 - 90.3) |  | 51.7%  (31.2 - 85.7) | 82.1%  (75.9 - 88.9) |  | 78.0%  (69.5 -87.6) | 81.5%  (72.5 - 91.6) |  |
