# Supplementary material for: Role of comorbidities and medication on immunotherapy efficacy in cSCC: A DeCOG multicentre analysis
Source: J Eur Acad Dermatol Venereol. 2026 Jan 29;40(9):1525–34. doi: 10.1111/jdv.70307 (PMC13505687; doi:10.1111/jdv.70307)

Supplementary Figure 6

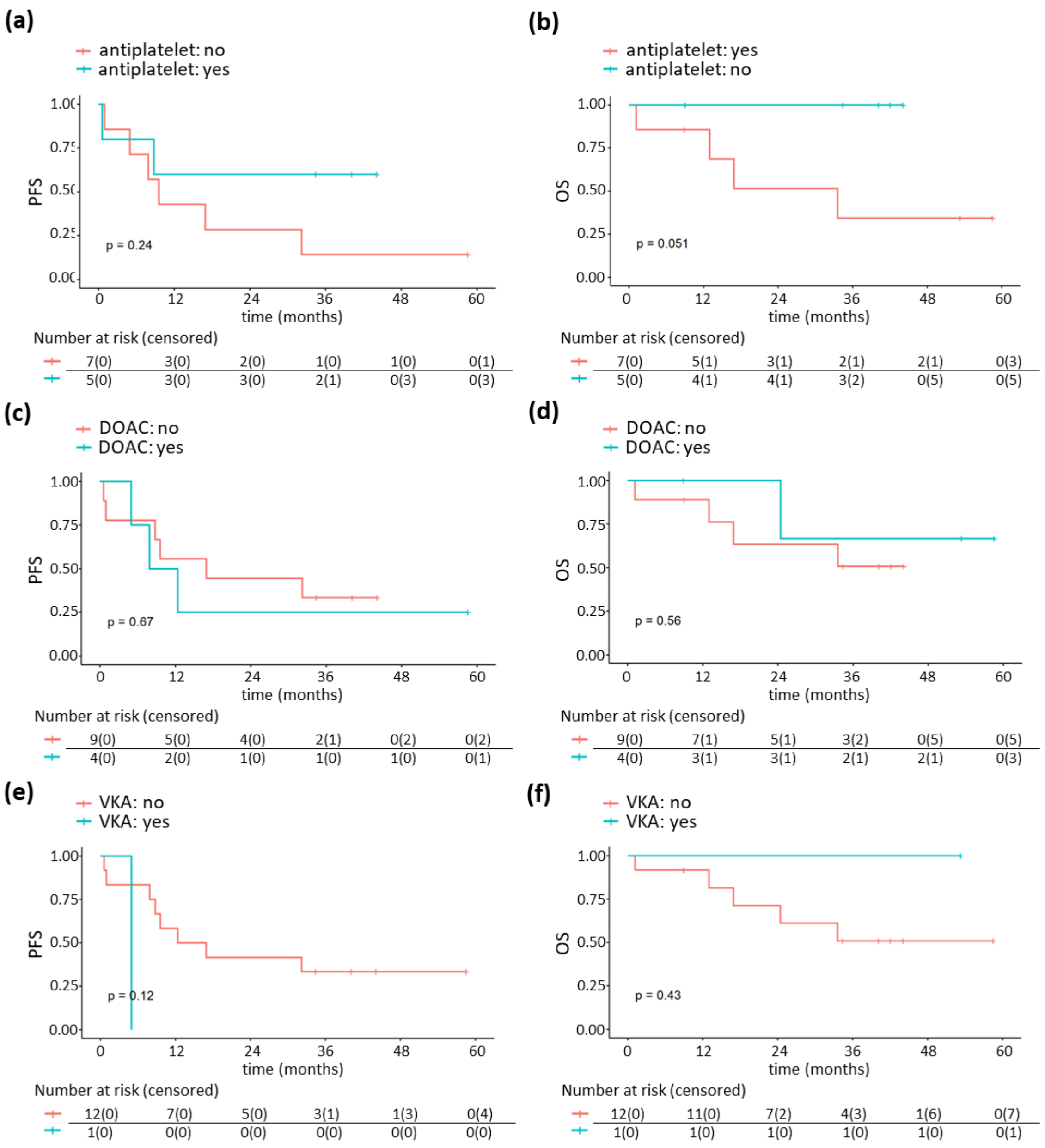

Supplement: Supplementary file 1 [file JDV-40-1525-s001.zip › jeadv-2025-2514-File014.pdf]
